# Treatment of human skeletal muscle cells with inhibitors of diacylglycerol acyltransferases 1 and 2 to explore isozyme-specific roles on lipid metabolism

**DOI:** 10.1038/s41598-019-57157-5

**Published:** 2020-01-14

**Authors:** Nils G. Løvsletten, Helene Vu, Christine Skagen, Jenny Lund, Eili T. Kase, G. Hege Thoresen, Victor A. Zammit, Arild C. Rustan

**Affiliations:** 1 Section for Pharmacology and Pharmaceutical Biosciences, Department of Pharmacy, University of Oslo, Oslo, Norway; 2 Department of Pharmacology, Institute of Clinical Medicine, University of Oslo, Oslo, Norway; 3 Division of Translational and Experimental medicine, Warwick Medical School, University of Warwick, Coventry, UK

**Keywords:** Biochemistry, Cell biology

## Abstract

Diacylglycerol acyltransferases (DGAT) 1 and 2 catalyse the final step in triacylglycerol (TAG) synthesis, the esterification of fatty acyl-CoA to diacylglycerol. Despite catalysing the same reaction and being present in the same cell types, they exhibit different functions on lipid metabolism in various tissues. Yet, their roles in skeletal muscle remain poorly defined. In this study, we investigated how selective inhibitors of DGAT1 and DGAT2 affected lipid metabolism in human primary skeletal muscle cells. The results showed that DGAT1 was dominant in human skeletal muscle cells utilizing fatty acids (FAs) derived from various sources, both exogenously supplied FA, *de novo* synthesised FA, or FA derived from lipolysis, to generate TAG, as well as being involved in *de novo* synthesis of TAG. On the other hand, DGAT2 seemed to be specialised for *de novo* synthesis of TAG from glycerol-3-posphate only. Interestingly, DGAT activities were also important for regulating FA oxidation, indicating a key role in balancing FAs between storage in TAG and efficient utilization through oxidation. Finally, we observed that inhibition of DGAT enzymes could potentially alter glucose–FA interactions in skeletal muscle. In summary, treatment with DGAT1 or DGAT2 specific inhibitors resulted in different responses on lipid metabolism in human myotubes, indicating that the two enzymes play distinct roles in TAG metabolism in skeletal muscle.

## Introduction

Skeletal muscle utilizes both carbohydrates and fat as energy sources. Approximately 50–60% of the free fatty acids (FFAs) taken up by skeletal muscle are stored as triacylglycerol (TAG) in lipid droplets (LDs)^1^. TAG, which is a neutral lipid, consists of a glycerol backbone and three FAs attached by ester bonds. The terminal and only committed step of TAG synthesis, the esterification of fatty acyl-CoA to diacylglycerol (DAG), is catalysed by the enzymes diacylglycerol acyltransferase (DGAT) 1 and 2^2–4^. Both DGAT enzymes reside at the endoplasmic reticulum^5,6^, though DGAT2 is also found to co-localize with LDs and mitochondria in cultured fibroblasts and adipocytes, in contrast to DGAT1^5,6^. Although the two isozymes catalyse the same reaction, there are several differences between them. They share no sequence homology with each other, belong to unrelated families of proteins^4^ and overexpression of the two isozymes in rat hepatoma cells give rise to LDs with markedly different morphology (size) and intracellular distribution^7^. In addition, they are non-redundant in some functions, which are reflected by the phenotype of mice lacking *DGAT1* or *DGAT2*. Whereas *Dgat1*^−/−^ mice are viable with a favourable metabolic phenotype showing an increased insulin and leptin sensitivity and resistance to diet-induced obesity, *Dgat2*^−/−^ mice die shortly after birth; they are lipopenic, have a defect in the skin barrier leading to rapid dehydration^8–10^, and are possibly unable to utilize glucose in brown adipocytes for thermoregulation^11^.

TAG formation can occur in two ways, namely from re-esterification of partial glycerides (derived from lipolysis of TAG) and through *de novo* incorporation of glycerol 3-phosphate into the glyceride entity followed by formation of DAG and TAG^12,13^. Several studies have been done to investigate and determine the roles of DGAT1 and DGAT2 in different tissues. For instance, the enzymes demonstrated to have non-redundant roles in intestinal lipid metabolism in mice enterocytes^14^. In liver and brown adipose tissue, DGAT1 seems to favour the incorporation of exogenous supplied FAs, whereas DGAT2 appears to be an enzyme of major importance for TAG synthesis of FAs derived from *de novo* lipogenesis^11,15,16^. Moreover, DGAT1 and DGAT2 have recently been shown to have distinct and overlapping functions for TAG synthesis in adipocytes^17^, where DGAT1 have been linked to the lipolysis-re-esterification cycle of preformed FA, a process that may also protect the endoplasmic reticulum from lipotoxic stress and adipose tissue inflammation^18^.

In muscle, almost all previous studies have focused on DGAT1. Human cardiomyocytes and cultured mouse myocytes treated with a specific DGAT1 inhibitor exhibited reduced mRNA expression of genes mediating FA uptake and oxidation^19^. Further, inactivation of *DGAT1* in a mouse cardiac model reduced TAG synthesis and increased FA oxidation, whereas co-inhibition of DGAT1/2 abrogated TAG synthesis and protected against high fat diet-induced lipid accumulation^20^. Interestingly, upregulation of *DGAT1* in mouse skeletal muscle increased TAG synthesis and protected against high-fat diet-induced insulin resistance^21^, whereas overexpression of *DGAT2* in glycolytic muscle resulted in an increased amount of TAG, ceramides and long-chain fatty acyl-CoAs, followed by an impaired insulin signalling^22^. Overall, these reports emphasize the potential for specialized roles of DGAT1 and DGAT2 in various tissues. Moreover, skeletal muscle is an important site for metabolic disturbances^23^ and the balance between storage and efficient utilization of TAG is a potential key to understand the interaction in dysregulated fat and glucose metabolism in skeletal muscle^24,25^.

In the present study we wanted to explore whether the roles of DGAT1 and DGAT2 are also specialized in human primary myotubes and to determine if DGAT1 and DGAT2 in skeletal muscle follow the same patterns of lipid incorporation that has previously been shown in other cell types. Using highly specific small-molecule inhibitors of DGAT1 (A922500, D1i)^26^ and DGAT2 (JNJ-DGAT2-A, D2i)^16^ we investigated the effects of their specific inhibition on TAG synthesis in FA metabolism using labelled precursors. Further, we examined the effect of DGAT1 and DGAT2 inhibition on other parameters including FA turnover (oxidation, lipolysis and re-esterification). Additionally, we examined if inhibition of DGAT enzymes also could influence glucose metabolism in human myotubes.

## Materials and Methods

### Materials

Dulbecco’s modified Eagle’s medium (DMEM-Glutamax) low glucose with sodium pyruvate, Dulbecco’s phosphate buffered saline (DPBS, without Mg^2+^ and Ca^2+^), foetal bovine serum (FBS), penicillin-streptomycin (10000 IE/ml), amphotericin B, Collagen I, Hoechst 33258, Bodipy 493/503, Pierce BCA Protein Assay Kit, Power SYBR Green PCR Master Mix, MicroAmp Optical Adhesive Film, MicroAmp Optical 96-well Reaction Plate and TaqMan Reverse Transcription Reagents were from ThermoFisher Scientific (Waltham, MA, US). Ultroser G was purchased from Pall Life Sciences (Cergy-Saint-Christophe, France). Insulin (Actrapid) was obtained from NovoNordisk (Bagsvaerd, Denmark). Bovine serum albumin (BSA, essentially FA-free), L-carnitine, D-glucose, oleic acid (OA, 18:1, n-9), HEPES, DMSO, gentamicin, glycogen, etomoxir, A922500, and β-mercaptoethanol were from Sigma-Aldrich (St. Louis, MO, US). T0901317 was purchased from Cayman Chemical Company (Ann Arbor, MI, US). [^14^C]oleic acid (OA, 56–59 mCi/mmol), D-[^14^C(U)]glucose (107.3 mCi/mmol and 263 mCi/mmol), D-[^14^C(U)]glycerol (142 mCi/mmol), and [^14^C]acetate (50.5 mCi/mmol) were purchased from PerkinElmer NEN (Boston, MA, US). 96-well and 6-well Corning CellBIND tissue culture plates were from Corning (Schiphol-Rijk, the Netherlands). 96-well Scintiplate tissue culture plates, UniFilter-96 GF/B microplates, Isoplate-96 scintillation microplates, TopSeal-A transparent film, OptiPhase Supermix, and Ultima Gold were obtained from PerkinElmer (Shelton, CT, US). TG PAP 150-kit was from Biomérieux (Craponne, France). Thin layer chromatography plates were purchased from Merck (Darmstadt, Germany). QIAshredder and RNeasy Mini kit were from QIAGEN (Venlo, the Netherlands). Glass Bottom Microwell Dishes (35 mm petri dish, 14 mm microwell, No. 1.5 cover glass) were obtained from MatTek (Ashland, MA, US). Bio-Rad Protein Assay Dye Reagent Concentrate was from Bio-Rad (Copenhagen, Denmark). JNJ-DGAT2-A was from Janssen Research and Development (High Wycombe, UK).

### Methods

#### Culturing of human myotubes

Multinucleated myotubes were established by activation and proliferation of myoblasts obtained from satellite cells. Satellite cells were isolated, proliferated to myoblast that were stored in a cell bank and further cultured as previously described^27^ from the *musculus vastus lateralis* of eight healthy young male subjects. Donors were 25.5 (±0.95) (mean ± SEM) years old with a body mass index of 22.1 (±0.8) kg/m^2^. More details about the donor cohort used in this study, as well as characteristics of myotubes can be found in the study by Lund *et al*.^28^. Muscle biopsies were obtained after informed written consent and approval by the National Committee for Research Ethics, Oslo, Norway (2011/2207 REK South East B). The study adhered to the Declaration of Helsinki.

For most experiments, the cells were cultured on multiwell plates in DMEM-Glutamax (5.5 mM glucose) supplemented with 2% FBS, 25 IU penicillin, 25 µg/ml streptomycin, 1.25 µg/ml amphotericin B, 50 ng/ml gentamycin, and 2% Ultroser G. At approximately 80% confluency, the growth medium was replaced by DMEM-Glutamax (5.5 mM glucose) supplemented with 2% FBS, 25 IU penicillin, 25 µg/ml streptomycin, 1.25 µg/ml amphotericin B, 50 ng/ml gentamycin, and 25 pM insulin. During culturing, the cells were incubated in a humidified 5% CO_2_ atmosphere at 37 °C, and the medium was changed every 2–3 days as previously described^29^. Experiments were performed on myotubes after 6–7 days of differentiation. Cells from all eight donors were not used in all experiments.

#### Lipid distribution – incorporation from OA and glycerol

The muscle cells were cultured on 12- or 24-well plates. After 6–7 days of differentiation, the myotubes were incubated with 100 µM [^14^C]OA (0.5 µCi/ml) in DMEM-Glutamax (5.5 mM glucose) supplemented with L-carnitine (1 mM) and BSA (40 µM) for 4 h. Alternatively, myotubes were given DMEM-Glutamax (5.5 mM glucose) supplemented with 10 µM D-[^14^C(U)]glycerol (1 µCi/ml), L-carnitine (1 mM) and BSA (40 µM) with or without 100 µM OA. DGAT1 inhibitor (D1i, A922500, 1 µM) or DGAT2 inhibitor (D2i, JNJ-DGAT2-A, 10 µM) were added to the cells 30 min before the radiolabelled substrate was added and incubated for 4 h. Thereafter, the myotubes were washed twice with 0.5 ml PBS, harvested in 125 µl distilled water and frozen at −20 °C. Cellular lipids were extracted and separated as described earlier^30^. Briefly, lipids were extracted by addition of chloroform:methanol (2:1, v/v) and 0.9% sodium chloride solution (pH 2). The mixture was then separated into two phases and the organic phase was dried under nitrogen gas and lipids were re-dissolved in hexane. Lipids were separated by thin layer chromatography (TLC), and radioactivity was quantified by liquid scintillation (Packard Tri-Carb 1900 TR, PerkinElmer, US). The amount of lipids was related to total cell protein content measured according to Bradford^31^. For lipid distribution we have done 3 or more individual experiments (*n*) with triplicate repetitions (3 separate culture wells) for each condition, see legends to figures.

#### Imaging

Myotubes were cultured on glass bottom microwell dishes coated with Collagen I (0.01%). At day 7 of differentiation, the cells were incubated with 100 µM OA for 4 and 24 h in the presence or absence of DGAT inhibitors (D1i, 1 µM; D2i, 10 µM). Myotubes were then incubated with Bodipy 493/503 (2 µg/ml) to stain lipid droplets, and Hoechst 33258 (2.5 µg/ml) to stain nuclei, both for 15 min. Cells were then washed with PBS and fixated with 3% paraformaldehyde for 20 min. Pictures were taken with an UPLSAPO 60x objective (NA:1.35) and images were taken in 6–8 positions in each well, using a confocal microscope (Olympus FLUOVIEW FV1000 confocal microscope, Japan). The calculated optical thickness was 0.7 µm for the whole image. Images were analyzed using ImageJ version 1.51j8 (NIH, US)^32^. Briefly, the number of lipid droplets were counted by ImageJ before nuclei were accounted for manually. If there were several nuclei in the edge, they were calculated together (two half nuclei equal one nucleus in the analysis), as long as they had surrounding lipid droplets confirmed by visual observation and ImageJ.

#### Determination of total TAG content

Myotubes grown in 75 cm^2^ cell culture flasks were incubated with 100 µM OA in presence or absence of D1i (1 µM) or D2i (10 µM) for 24 h. Thereafter, the myotubes were washed with PBS and harvested in 0.1% SDS. Measurement of total cellular TAG content after extraction, separation by TLC and redissolution in 50 μl 2-propanol, was performed with the TG PAP 150-kit according to the supplier’s protocol (Biomérieux). Basal TAG content in myotubes without OA was approximately 30 µg/mg protein, and after 24 h with 100 µM OA TAG content increased 2–3-fold.

#### Lipid distribution - incorporation from acetate and glucose

Myotubes were cultured on 12-well tissue culture plates. After 6–7 days of differentiation, the myotubes were incubated in presence or absence of D1i (1 µM) or D2i (10 µM) together with 100 µM [^14^C]acetate (2 µCi/ml) in DMEM-Glutamax (5.5 mM glucose) supplemented with BSA (40 µM) for 4 h. Thereafter, cells were harvested and cellular lipids extracted and quantified as described under section “Lipid distribution”. Alternatively, myotubes were treated with liver X receptor agonist T0901317 (1 µM) the last 4 days of differentiation to promote FA synthesis^33^. Thereafter the cells were incubated for 24 h in DMEM-Glutamax (5.5 mM glucose) supplemented with D-[^14^C(U)]glucose (2 µCi/ml), 2% FBS and 25 pmol/l insulin in presence or absence of D1i or D2i. Then, cells were harvested and quantified as described above.

#### Substrate oxidation assay and acid soluble metabolites for OA

Myotubes were cultured and treated as described above (sections “Culturing of human myotubes” and “Lipid distribution”). After 4 h of incubation with [^14^C]OA (0.5 µCi/ml, 100 µM) in presence of D1i (1 µM) or D2i (10 µM), 100 µl (24-well) or 200 µl (12-well) of radiolabelled cell medium was transferred to a multiwell plate, sealed and frozen at −20 °C. To measure CO_2_ production, 40 µl of 1 M perchloric acid (HClO_4_) was added immediately to the frozen medium. A 96-well UniFilter-96 GF/B microplate was activated to capture CO_2_ by addition of 20 µl 1 M sodium hydroxide and was mounted on top of the multiwell plate as previously described^34^. The mixture was then incubated at room temperature for 3 h to trap radiolabelled CO_2_. The CO_2_ produced during the 4 h of incubation with [^14^C]OA was captured by the sodium bicarbonate buffer system in the cell medium. After adding HClO_4_ to the frozen medium, CO_2_ was released and captured in the UniFilter-96 GF/B microplate. Radioactivity was measured by liquid scintillation (2450 MicroBeta^2^ scintillation counter, PerkinElmer).

Measurement of acid soluble metabolites (ASMs), which reflects incomplete FA oxidation (β-oxidation) and consists mainly of tricarboxylic acid cycle metabolites, was performed using a method modified from Skrede *et al*.^35^. From the radiolabelled incubation medium, 100 µl was transferred to a new Eppendorf tube and precipitated with 300 µl cold HClO_4_ (1 M) and 30 µl BSA (6%). Then, the tube was centrifuged at 10 000 rpm/10 min/4 °C, before 200 µl of the supernatant was counted by liquid scintillation (Packard Tri-Carb 1900 TR, PerkinElmer).

#### Scintillation proximity assay (SPA) and lipolysis assay

SPA is a method to measure the amount of radiolabelled substrates inside the cells. The scintillator is embedded in the bottom of each well and provides a stronger signal when radiolabelled substrates are accumulated in the cells compared to in the medium alone^34^. Human myotubes were cultured on 96-well ScintiPlate tissue culture plates. After 6 days of differentiation the cells were given DMEM without phenol red supplemented with [^14^C]OA (0.5 µCi/ml, 100 µM), 5.5 mM glucose, 2% FBS, 25 IU penicillin, 25 µg/ml streptomycin, 1.25 µg/ml amphotericin B, and 25 pM insulin. After 24 h the cells were washed twice with DPBS with 0.5% BSA, before fresh DPBS medium supplemented with 0.5% BSA, 10 mM HEPES and 0.1 mM glucose were added to the cells. The decline in [^14^C]OA in the cells (taken as a measurement of lipolysis^36^) were measured after 0, 1, 2, 4, and 6 h in presence or absence of DGAT inhibitors (D1i, 1 µM; D2i, 10 µM) and etomoxir (10 µM). Etomoxir is a selective inhibitor of mitochondrial carnitine palmitoyltransferase-1, thereby inhibiting FA oxidation by 90% at 10 µM^34,37^. Remaining cell-associated radioactivity was measured by liquid scintillation.

#### Substrate oxidation assay for glucose

Myotubes were cultured on 96-well tissue culture plates. At day 7 of differentiation, cells were given [^14^C(U)]glucose (0.5 µCi/ml, 200 µM) with D1i (1 µM) or D2i (10 µM) during 4 h of CO_2_ trapping as previously described^34^. CO_2_ production was measured in DPBS medium with 10 mmol/l HEPES, 1 mmol/l L-carnitine and 10 µM BSA. CO_2_ and cell-associated labelled glucose were measured by liquid scintillation using a 2450 MicroBeta^2^ scintillation counter (PerkinElmer).

#### Glycogen synthesis

Myotubes were cultured on 12-well tissue culture plates as described under section “Culturing of human myotubes”. At day 7 of differentiation, the myotubes were given DMEM medium without glucose for 90 min to starve the cells. Then, the myotubes were incubated with D-[^14^C(U)]glucose (1 µCi/ml, 5.5 mM) in presence or absence of D1i (1 µM) or D2i (10 µM) and with or without insulin (100 nM) for 3 h. Thereafter, the cells were washed twice with PBS and harvested in 1 M potassium hydroxide (KOH). Protein content was measured by Pierce BCA Protein Assay Kit. D-[^14^C(U)]glucose incorporated into glycogen was measured as previously described^38^ with some modifications. Briefly, glycogen and more KOH were added to the samples for a final concentration of 20 mg/ml and 19%, respectively. The samples were incubated for 20 min at 80 °C, before glycogen were precipitated in the samples by adding 100% ice-cold ethanol and centrifuged at 10 000 rpm for 20 min at 4 °C. The pellets were washed once with ice-cold 70% ethanol, centrifuged at 10 000 rpm for 20 min at 4 °C, air-dried, and re-suspended in distilled water. Radioactivity was measured by liquid scintillation (Packard Tri-Carb 1900 TR, PerkinElmer) and the amount of glycogen was related to total cell protein content.

#### Presentation of data and statistics

Data are presented as mean ± SEM if not stated otherwise, as absolute values and as % of control, where data are normalized to the mean value for the control within each separate experiment. The value *n* represents number of individual cell culture experiments normally with 3–8 cell culture wells for each condition in each experiment unless specified otherwise in figure legends. Statistical analyses were performed using GraphPad Prism 7.02 Software (GraphPad Software Inc., La Jolla, CA, US), where two-tailed paired t-test was performed to determine effects of treatment unless specified otherwise in figure legends. The number of experiments (*n*) was increased for some parameters studied when it was expected to observe small treatment effects. Linear mixed-model analysis (LMM, SPSS 25.0.0.1, IBM SPSS Inc., Chicago, IL, US) was used in the time-course lipolysis experiments (SPA). p < 0.05 was considered significant.

## Results

### Effect of DGAT inhibitors on distribution of exogenous OA in various lipid classes

We first wanted to explore the roles of DGAT1 and DGAT2 in metabolism of exogenously added [^14^C]oleic acid (OA), with particularly emphasis on TAG synthesis in human myotubes. Initially, we confirmed that both DGAT enzymes were expressed in human myotubes, where *DGAT1* appeared to be most abundant (Fig. S-1). To determine the optimal concentrations of the specific inhibitors of DGAT1 (A922500, D1i, IC_50_ value of 7 nM)^26^ and DGAT2 (JNJ-DGAT2-A, D2i, IC_50_ value of 140 nM)^16^, myotubes were treated with labelled OA and different concentrations of the inhibitors. Two processes were measured: incorporation and complete oxidation of [^14^C]OA into TAG and CO_2_, respectively (Fig. S-2). Based on these results and available literature^11,16,36^, concentrations for further experiments were determined to be 1 µM (D1i) and 10 µM (D2i). Furthermore, visual observation and measurement of cell protein content indicated none cell-toxic effects with neither D1i (1 µM) nor D2i (10 µM) up to 24 h treatment. Additionally, acute treatment with DGAT inhibitors did not influence expression of several genes involved in lipid metabolism (FA oxidation, storage, lipolysis, and turnover) in human myotubes (Fig. S-3).

**Figure 1:**
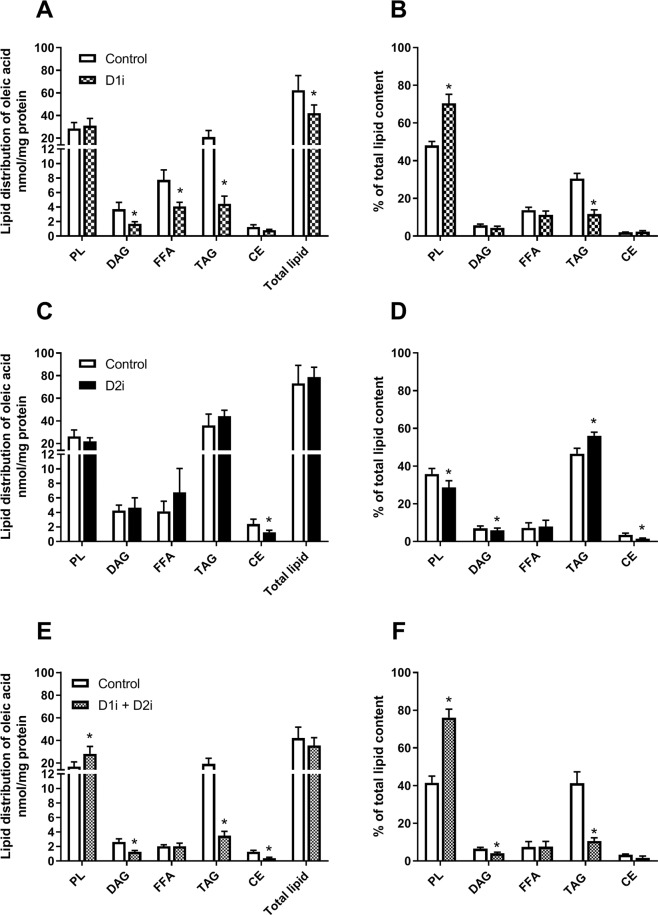
Effect of DGAT inhibitors on incorporation of oleic acid into various lipid classes. Human myotubes were grown and differentiated on 12- or 24-well tissue culture plates for 7 days. On day 7 of differentiation myotubes were incubated with 100 µM [^14^C]oleic acid (0.5 µCi/ml) for 4 h in presence or absence of D1i (1 µM) and/or D2i (10 µM). Cellular lipids were extracted and separated by thin layer chromatography (TLC), and radioactivity was measured by liquid scintillation. The sum of all radioactivity on the TLC plate is presented as total lipids. (**A–F**) Lipid distribution of [^14^C]oleic acid after treatment with D1i, D2i and combination of D1i and D2i. Results are presented as mean ± SEM as nmol/mg protein (**A,C,E**) or as % of total lipids in the cell (**B,D,F**). *n* = 6 (**A,B**), *n* = 8 (**C,D**) and *n* = 5 (**E,F**) individual experiments with 3 separate culture wells for each condition. *p < 0.05 vs. control, paired t-test. CE, cholesteryl ester; D1i, DGAT1 inhibitor; D2i, DGAT2 inhibitor; DAG, diacylglycerol; FFA, free fatty acid; PL, phospholipid; TAG, triacylglycerol.

We aimed to characterize how acute treatment with DGAT inhibitors influenced lipid distribution of OA in the myotubes. Treatment with D1i significantly decreased the level of total lipid generated from [^14^C]OA. D1i reduced the levels of TAG by ~80% compared to control, while diacylglycerol (DAG) and FFAs were reduced to a lesser extent (Fig. 1A**)**. The highest proportion of total lipids was recovered in phospholipids (PLs) and TAG, which showed an increased and decreased percentage after treatment with D1i, respectively (Fig. 1B). Treatment with D2i had little effect on absolute values, with only a minor reduction in the levels of cholesteryl ester (CE) (Fig. 1C). However, when calculated as percentage of total lipids, it was a paradoxical increase in the percentage of TAG, accompanied by decreased levels of PLs, DAG and CE (Fig. 1D). When combining the two inhibitors the effect on TAG reduction was similar to D1i alone (~80%). Further, D1i + D2i increased the levels of PLs, and reduced the levels of CE, DAG and TAG (Fig. 1E). A substantially increased percentage of total lipids was recovered as PLs, whereas the fraction of DAG and TAG was decreased, indicating a shift in the flux of OA from DAG and TAG into PLs (Fig. 1F).

**Figure 2:**
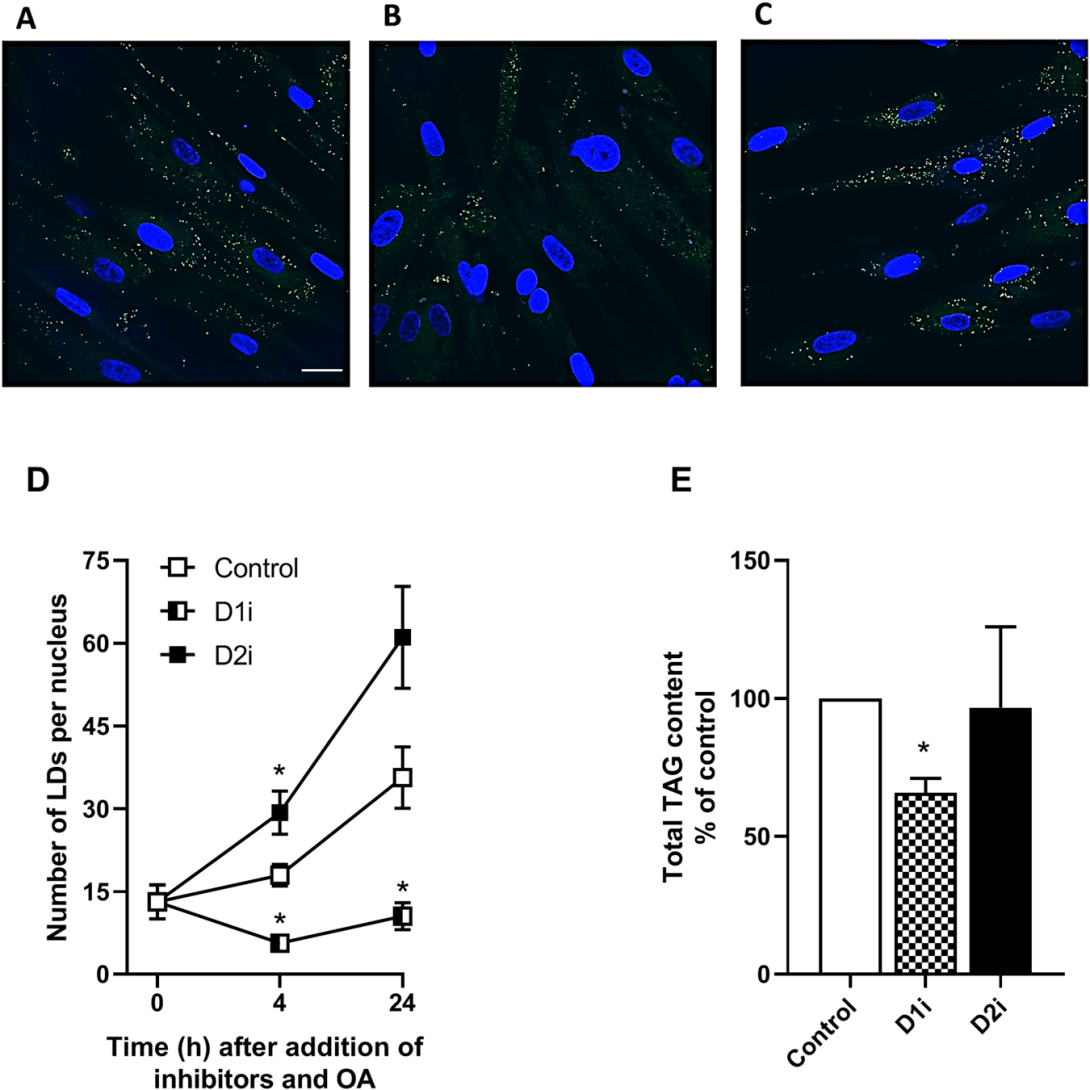
Effect of DGAT inhibition on lipid droplets and total cellular triacylglycerol content. Human myotubes were grown and differentiated on glass bottom microwell dishes. At day 7 of differentiation, myotubes were incubated with 100 µM OA for 4 (**A–C**) and 24 h (**D**) in presence or absence of DGAT inhibitors; D1i (1 µM) and D2i (10 µM). The cells were stained for lipid droplets (green) and nuclei (blue) using Bodipy 493/503 and Hoechst 33528, respectively, and images taken with a 60x objective on a confocal microscope. Scale bar 25 µm. (**A–C**) Representative images are presented for control (**A**), D1i (**B**) and D2i (**C**). (**D**) LDs were quantified (ImageJ) by relating number of LDs to number of nuclei. Results represent mean ± SEM from one experiment at 4 h and one experiment at 24 h where calculations are based on 6–8 different images for each condition, unpaired t-test, *p < 0.05 vs control at the same time-point. (**E**) Human myotubes were incubated with 100 µM OA in presence or absence of D1i (1 µM) and D2i (10 µM) for 24 h and total TAG content was measured. The results are presented as mean ± SEM as % of control from *n* = 3 individual experiments with one 75 cm^2^ cell culture flask for each condition. *p < 0.05 vs. control, paired t-test. D1i, DGAT1 inhibitor; D2i, DGAT2 inhibitor; LDs, lipid droplets; OA, oleic acid; TAG, triacylglycerol.

The differences induced by DGAT inhibitors on incorporation of OA into various lipid classes were reflected in changes in the number of LDs after incubation with OA and either of the two inhibitors (Fig. 2). Treatment with D1i reduced the number of LDs, whereas the number of LDs in cells treated with D2i was slightly increased compared to the control cells (Fig. 2D). Furthermore, a 35% reduction in total TAG content was seen in the myotubes after 24 h treatment with D1i and OA (Fig. 2E).

### Effect of DGAT inhibitors on incorporation of glycerol, acetate and glucose into TAG and total lipids

**Figure 3:**
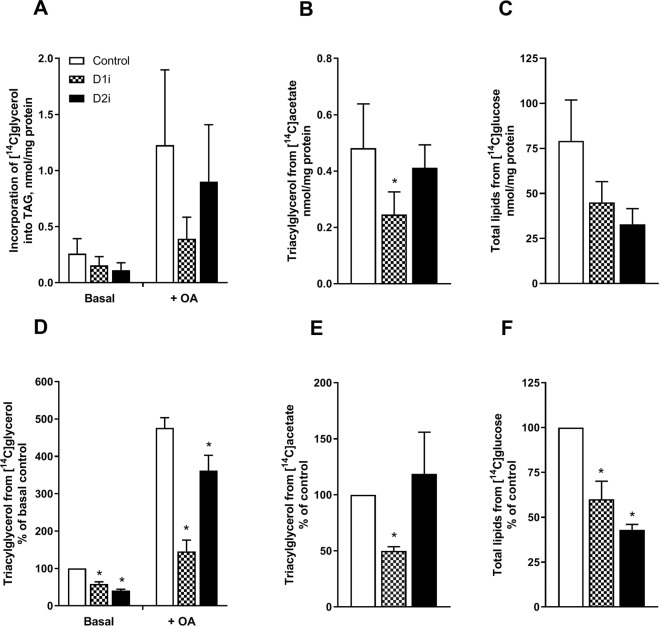
Effect of DGAT inhibition on incorporation of glycerol, acetate and glucose into TAG and total lipids. Human myotubes were grown and differentiated on 12-well tissue culture plates for 6–7 days. At day 7 of differentiation myotubes were incubated with D-[^14^C(U)]glycerol (1 µCi/ml, 10 µM) supplemented with D1i (1 µM) or D2i (10 µM), in presence or absence of 100 µM oleic acid for 4 h (**A,D**) or with 100 µM [^14^C]acetate (2 µCi/ml) in presence or absence DGAT inhibitors for 4 h (**B,E**). **C,F**) Cells were incubated with the liver X receptor agonist T0901317 (1 µM) for 96 h. Thereafter, myotubes were incubated for 24 h with D-[^14^C(U)]glucose (2 µCi/ml, 5.5 mM) in presence or absence of DGAT inhibitors. Lipids were separated by thin layer chromatography and measured using liquid scintillation. The sum of all radioactivity on the TLC plate is presented as total lipids. Results represent mean ± SEM from *n* = 4 (**A,B**), *n* = 5 **(D,E**) or *n* = 3 (**C,F**) individual experiments with 3 separate culture wells for each condition presented at absolute values (**A, B, C**) or normalized to control (**D–F**). *p < 0.05 vs. control, paired t-test. D1i, DGAT1 inhibitor; D2i, DGAT2 inhibitor; TAG, triacylglycerol.

We next wanted to study the incorporation of a substrate involved in *de novo* synthesis of TAG, *i.e*. glycerol. Therefore, myotubes were incubated with D-[^14^C(U)]glycerol with or without OA for 4 h in presence or absence of D1i or D2i. Both in absence and presence of OA, D1i and D2i had an inhibiting effect on TAG synthesis measured by incorporation of [^14^C]glycerol. Interestingly, D2i appeared to reduce the TAG levels to a greater extent (~40% vs. ~60%) in the absence of OA, whereas D1i reduced TAG levels most strikingly in the presence of OA (~70% vs. 25%) (Fig. 3A,D).

To examine lipid utilization for TAG synthesis in more detail, we used [^14^C]acetate as a marker for *de novo* synthesized FAs. Exposure to labelled acetate showed an inhibiting effect on TAG levels after treatment with D1i (Fig. 3B,E). Also, incorporation of acetate into phospholipids was decreased by DGAT1 inhibition (Supplemental Fig. S-4). Moreover, we also used [^14^C]glucose as substrate for incorporation of ^14^C-label into lipids. From experience we know that incorporation of [^14^C]glucose into cellular lipids in myotubes is usually low, but treatment with the liver X receptor agonist T0901317 has been shown to increase incorporation of [^14^C]glucose into lipids in this cell model^33,39^. Myotubes were therefore incubated with the liver X receptor agonist T0901317 (1 µM) for 96 h and then incubated for another 24 h with [^14^C]glucose in the presence or absence of DGAT inhibitors. Incorporation of [^14^C]glucose into labelled total lipids was significantly reduced for both D1i and D2i (Fig. 3C,F).

### Effect of DGAT inhibitors on lipid turnover and oxidation

**Figure 4:**
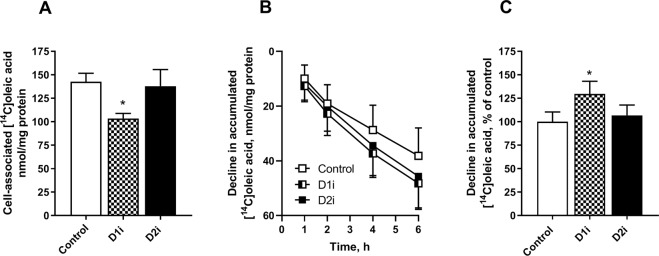
Effect on DGAT inhibition on turnover of accumulated lipids. Human myotubes were grown and differentiated on 96-well tissue culture plates, SPA plates were used for time-course (**B,C**). At day 6 of differentiation myotubes were incubated with [^14^C]oleic acid (0.5 µCi/ml, 100 µM) for 24 h. After 24 h pre-treatment with [^14^C]oleic acid myotubes were washed and re-incubated with D1i (1 µM) or D2i (10 µM). (**A**) Cell-associated radioactivity from [^14^C]oleic acid was measured after 4 h treatment with DGAT inhibitors. (**B,C**) Decline in cell-associated radioactivity was measured over 6 h in presence of an inhibitor of carnitine palmitoyltransferase 1 (etomoxir, 10 µM) and DGAT inhibitors; D1i (1 µM) and D2i (10 µM). Results represent mean ± SEM as nmol/mg protein (**A,B**) and as all-over effects normalized to control (**C**) from *n* = 4 individual experiments with 8 separate culture wells for each condition. *p < 0.05 vs control, paired t-test (**A**), LMM statistical test (**C**). D1i, DGAT1 inhibitor; D2i, DGAT2 inhibitor; SPA; scintillation proximity assay; LMM, Linear mixed model.

**Figure 5:**
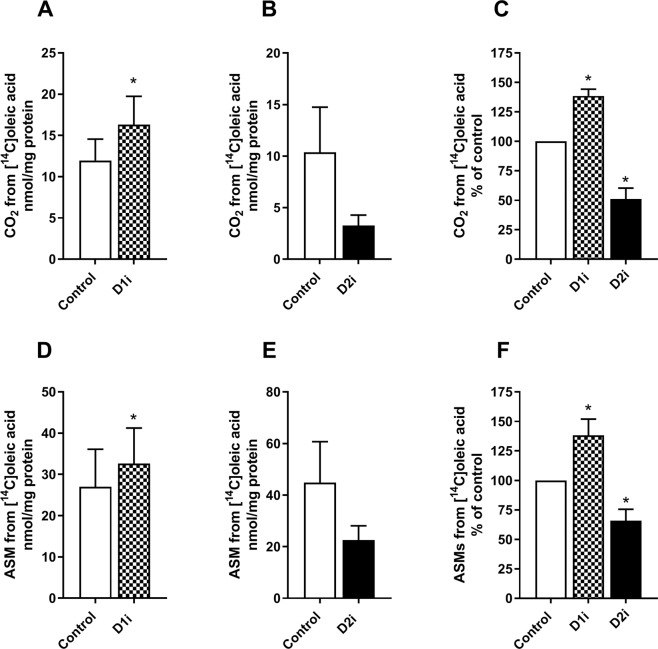
Effect of DGAT inhibition on oleic acid oxidation. Human myotubes were grown and differentiated on 12- or 24-well tissue culture plates. At day 7 of differentiation, myotubes were incubated with 100 µM [^14^C]oleic acid (0.5 µCi/ml, 100 µM) for 4 h in presence or absence of D1i (1 µM) or D2i (10 µM), respectively. Complete oxidation (CO_2_ production) and β-oxidation (ASMs) were measured. (**A–C)** Complete oxidation (CO_2_) of oleic acid. (**D–F)** ASMs, which reflects incomplete oxidation (β-oxidation), of oleic acid. Results are presented as mean ± SEM from *n* = 5–6 (D1i) and *n* = 8 (D2i) individual experiments with 8 separate wells for each condition, as absolute values (nmol/mg protein) (**A,B** and **D,E**) and normalized to control (**C,F**). *p < 0.05 vs control, paired t-test. ASMs, acid soluble metabolites; D1i, DGAT1 inhibitor; D2i, DGAT2 inhibitor.

Since DGAT inhibition was shown to have different effects on incorporation of radioactive labelled ^14^C from OA and glycerol into TAG, depending on which DGAT isozyme being inhibited, we further examined the roles of DGAT1 and DGAT2 on lipid turnover of accumulated lipids. We pretreated human myotubes with [^14^C]OA for 24 h to prelabel the endogenous TAG pools. After 24 h of incubation, fresh medium containing D1i or D2i was added to the cells. Cell-associated radioactivity (presumed to be mostly ^14^C-TAG or ^14^C-PL, see Fig. 1) was measured 4 h after adding the inhibitors. Treatment with D1i for 4 h reduced the levels of cell-associated ^14^C-labelled lipids compared to control. By contrast treatment with D2i showed no effect (Fig. 4A). When the experiments were performed in the presence of etomoxir (inhibitor of FA oxidation) during 6 h (Fig. 4B), cell-associated lipids were reduced in D1i-treated cells compared to control cells, indicating that only alterations in the rate of re-esterification were involved (Fig. 4C). Next, we examined the effects of the inhibitors on oxidation of [^14^C]OA, where both CO_2_ production (complete FA oxidation Fig. 5A–C) and acid-soluble metabolites (ASMs, FA β-oxidation, Fig. 5D–F) were measured. We observed a significant increased oxidation (ASMs and CO_2_) in cells treated with D1i, whereas treatment with D2i showed a reduced oxidation (ASMs and CO_2_).

### Effect of DGAT inhibitors on glucose metabolism

**Figure 6:**
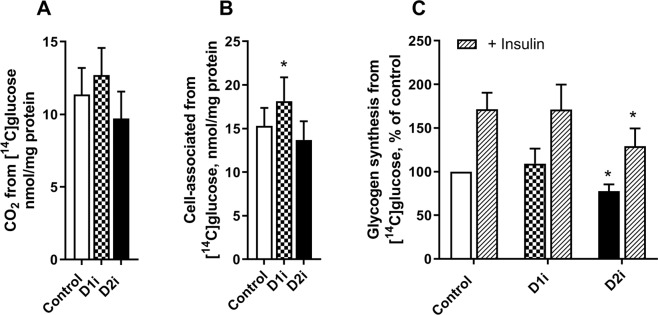
Effect of DGAT inhibitors on glucose metabolism. (**A–B**) Human myotubes were grown on 96-well tissue culture plates. At day 7, myotubes were incubated with [^14^C(U)]glucose (0.5 µCi/ml, 200 µM) and D1i or D2i for 4 h. Oxidation, measured as CO_2_ production from [^14^C(U)]glucose (**A**) and cell-associated radioactivity from [^14^C(U)]glucose (**B**) was measured after 4 h treatment with DGAT inhibitors; D1i (1 µM) and D2i (10 µM). (**C**) Alternatively, cells were grown in 12-well tissue culture plates. At day 7, myotubes were starved for 90 min (DMEM without glucose) before incubation for 3 h with D-[^14^C(U)]glucose (1 µCi/ml, 5.5 mM) in presence or absence of D1i (1 µM) or D2i (10 µM) and with or without insulin (100 nM). Results are presented as mean ± SEM from *n* = 9 (**A,B**) individual experiments with 8 separate wells for each condition, or *n* = 4 (**C**) individual experiments with duplicate wells for each condition. Absolute values for glycogen synthesis: control 11.0 ± 4.5 and with insulin 22.4 ± 10.8 nmol/mg cell protein. *p < 0.05 vs control, paired t-test.

Skeletal muscle is a major consumer of both FAs and glucose, and shows a high metabolic flexibility. Since DGAT enzymes play a central role in lipid metabolism, and our data also indicated the importance of DGAT in determining the flux of FAs to oxidation as well as storage (Fig. 5), we wanted to study possible effects of acute inhibition of DGAT enzymes on glucose metabolism in human myotubes. Although there was no effect of the DGAT inhibitors on glucose oxidation, we observed a small but significant increased glucose uptake after treatment with D1i (Fig. 6A,B). Moreover, treatment with D2i reduced the level of basal as well as insulin-stimulated glycogen synthesis compared to control (Fig. 6C).

## Discussion and Conclusion

### Discussion

In this study, we present a description of the respective roles of DGAT1 and DGAT2 on lipid metabolism in primary human skeletal muscle cells. It was found that inhibition of DGAT1 and DGAT2 had distinct effects on lipid metabolism in skeletal muscle cells. Through the use of specific inhibitors, DGAT1 was shown to be the major enzyme responsible for incorporation of both exogenously supplied and endogenously generated (through lipolysis) FAs into cellular lipids in human myotubes. In addition, FAs derived from *de novo* FA synthesis were utilized by DGAT1. In contrast, DGAT2 could not compensate for the loss of DGAT1 in synthesizing TAG from exogenous or endogenous FAs generated by lipolysis. However, results indicated that DGAT2 played a prominent role in *de novo* synthesis of TAG from glycerol-3-phosphate at lower substrate concentrations. Similarly, DGAT1 contributed to this process to a greater extent when the concentrations of substrates were higher. Interestingly, inhibition of the two DGATs affected FA oxidation differently and so it would appear that DGAT activities are important in determining the rates of FA oxidation. Finally, we observed possible roles of DGATs on glucose accumulation and glycogen synthesis.

Skeletal muscle utilize FAs which are mainly metabolised through oxidation, TAG synthesis (storage in LDs) and PL synthesis^40^. We observed that treatment with D1i or D2i gave distinct responses on the handling of preformed FAs. D1i reduced the levels of TAG by nearly 80%, whereas D2i did not show any reducing effect on TAG synthesis when using exogenous FAs as substrate (Fig. 1). Instead, we observed a relative increased proportion of TAG using D2i. Thus, whereas inhibition of DGAT1 reciprocally lowered relative flux into TAG synthesis and increased flux into PLs, inhibition of DGAT2 appeared to achieve the opposite and induced a shift in the flux of preformed FAs from PLs into TAG. This might be catalysed by DGAT1, which is still active as it could be a competition between these two pathways for a common DAG substrate. The same pattern was reflected in LDs and total TAG content generated from exogenous added FAs, where only D1i reduced the quantity of LDs and TAG (Fig. 2). The lack of effect of D2i on TAG synthesis and LD formation from exogeneous fatty acid may be related to much lower expression of this enzyme compared to DGAT1. Our data from skeletal muscle cells is consistent with studies done in cells from other tissues, hepatocytes and brown adipocytes, which show that incorporation of exogenous FA into TAG is predominantly mediated by DGAT1, and not DGAT2^11,15,16^. However, the enzymes have been found to partially compensate for each other for TAG storage in adipocytes^17,18^. We observed that D1i almost totally abolished incorporation of exogenous FAs into TAG and LDs (Fig. 2), which demonstrate the major role of DGAT1 for overall TAG content in muscle cells, which further is supported by DGAT1 being most abundant in skeletal muscle^2^. This essential role of DGAT1 is compatible with studies done in murine skeletal muscle that show increased intramyocellular lipid content and TAG synthesis after overexpression of *DGAT1*^21,41,42^. Moreover, inactivation of *DGAT1* in heart resulted in a moderate suppression of TAG synthesis and turnover^20^. Also, exercise has been shown to induce the expression of DGAT1, followed by increased TAG levels in the muscles of mice and humans^21,43,44^. Thus, DGAT1 function has been linked to the “athlete paradox”, where endurance-trained athletes, in contrast to sedentary subjects, are highly insulin sensitive despite high levels of intramyocellular TAG^21^.

Myotubes were incubated with glycerol, to label the backbone of the glycerolipids in order to distinguish between the formation of TAG by re-esterification of partial glycerides and *de novo* synthesis of TAG (Fig. 3). Labelled acetate was used for measuring incorporation of FAs derived from *de novo* FA synthesis, while glucose could be incorporated in both the glycerol backbone and Fas (Fig. 3). Interestingly, we observed a reduction of TAG by both D1i and D2i when glycerol or glucose was used as label. The effect of D2i was most striking without OA present, with a ~60% reduction in labelled TAG, whereas in the presence of OA a minor reduction (~25%) was observed. In contrast, treatment with D1i showed a marked inhibition of glycerol incorporation into TAG (~70%) in presence of OA, compared to without (~40%). These data suggest, as supported by studies in other tissues^15,16^, that DGAT2 in skeletal muscle presumably contributes to *de novo* synthesis of TAG. Moreover, DGAT2 but not DGAT1 is co-localized with lipogenic enzymes like glycerol-3-phosphate acyltransferase and stearoyl-CoA desaturase 1^45,46^. Noteworthy, in the presence of exogenous FAs D1i was most effective in blocking TAG synthesis, even though the effect of D2i remained. This might be explained by substrate concentrations, as it has been reported that DGAT1 tends to be more active at higher substrate concentrations utilizing exogenous FA, whereas DGAT2 tends to be more active at lower FA concentrations^4^. Furthermore, DGAT2 has been suggested in some, but not all reports, to preferentially utilize endogenously synthesized FAs to form TAG in liver cells and brown adipocytes^11,15,16,47^. Our results did not indicate such a role of DGAT2 in skeletal muscle, where only DGAT1 inhibition reduced TAG synthesis from acetate, *i.e*. *de novo* FA synthesis. Also, incorporation of acetate into phospholipids was decreased by DGAT1 inhibition indicating that *de novo* FA synthesis was reduced when DGAT1 was inhibited, not only TAG synthesis (Fig. S-4). FA synthesis is, however, a complex process to measure in human primary muscle cells, since FA synthesis generally is limited^48^, and time-aspects, substrate availability and experimental conditions may play important roles.

Skeletal muscle is central for utilizing lipids for energy production^49^. FAs stored as TAG in LDs are easily hydrolysed upon energy demand, and thereby are an important energy source^25^. Furthermore, turnover of TAG in LDs reflects the balance between lipolysis and esterification^50^. Our data showed that treatment with D1i, but not D2i, reduced the accumulation of [^14^C]OA in cell lipids, and increased net lipolysis (Fig. 4). If it is assumed that the rate of lipolysis was the same between control and D1i-treated cells, there appears to have been a lower rate of re-esterification of FAs when DGAT1 was inhibited. Further, these data suggest that only DGAT1 is involved in the re-esterification of FA products of lipolysis in skeletal muscle. This is consistent with a previous observation in adipocytes, where DGAT1 has been shown to be the major enzyme involved in the re-synthesis of FAs derived from adipocyte lipolysis^18^. In our study we also observed an increased oxidation (complete and incomplete) after treatment with D1i, whereas D2i reduced both processes (Fig. 5). Increased oxidation after treatment with D1i confirmed previous observations, using the same D1i (1 µM) in human myotubes^36^. In accordance with this, a study in a cardiac-specific DGAT1 deletion mouse model showed an increased oxidation of exogenous FAs compared to wildtype^20^. We hypothesise that the increased oxidation we observed when DGAT1 was inhibited, is due to the diversion of FAs for oxidation. The reduced oxidation by D2i is on the other hand difficult to explain. It appears to be due to reduced substrate availability since both CO_2_ and ASMs showed a similar reduction. This indicates a possible competition between utilization of oleoyl-CoA by carnitine palmitoyltransferase 1 and/or mitochondrial glycerol-3-phosphate acyltransferase and DGAT1. Moreover, DGAT2, but not DGAT1, is associated with LDs and co-localized to mitochondria in cultured fibroblasts and adipocytes^5,6^, which may add a further dimension to the relationship between DGAT1 or DGAT2 activity and oxidation of exogenous or endogenous FAs.

As glucose and FA metabolism in skeletal muscle are tightly regulated^51,52^, we also examined the effect of DGAT inhibitors on glucose metabolism (Fig. 6). Inhibition of DGAT enzymes could potentially alter glucose–FA interactions, as DGAT1 and DGAT2 are catalysing synthesis of TAG, which is the primary unit for storage of FAs and also has the potential of protecting cells from deleterious lipid intermediates^53^. We did not observe any effect on glucose oxidation by either of the DGAT inhibitors. However, treatment with D1i increased accumulation of glucose, whereas D2i decreased basal and insulin-stimulated glycogen synthesis. Even though these effects were relatively small, this indicates a link between DGAT-regulated processes and glucose metabolism in skeletal muscle. We also examined glucose metabolism with DGAT inhibitors in presence of oleic acid added acutely (that was shown to increase or decrease FA oxidation in Fig. 5), but no changes were found on glucose metabolism nor insulin action (data not shown). In support of our observations treatment with JTT-553 (DGAT1 inhibitor) in mice resulted in increased glucose uptake in adipose tissue^54^. Moreover, DGAT2 has been linked to the generation of acylceramides; its inhibition may raise intracellular ceramide levels^55^. Thus, inhibition of DGAT2 could potentially affect ceramide signalling. We also examined the effect of DGAT inhibitors on acylceramide formation from labelled FA which was very low and not changed by the inhibitors (data not shown). Although interesting, these data on glucose metabolism are presently difficult to interpret and requires more elaborate studies that are outside the scope of the present study.

### Conclusion

**Figure 7:**
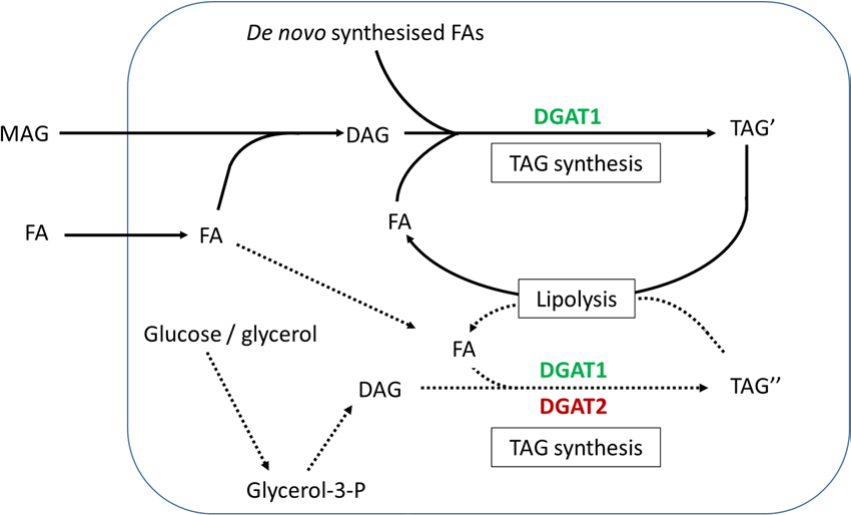
Model illustrating possible functions of DGAT1 and DGAT2 in human skeletal muscle cells. DGAT1 is dominant in human skeletal muscle cells utilizing FAs derived from various sources (exogenously supplied, *de novo* FA synthesis or FA derived from lipolysis) to generate TAG, both through the monoacylglycerol pathway and the *de novo* pathway from glycerol-3-P. DGAT2 seems to be specialised only for the synthesis of TAG, involving *de novo* incorporation of the glycerol moiety into TAG. Moreover, DGAT1 seems to operate at higher substrate concentrations, whereas DGAT2 may esterify substrates at lower concentrations for storage in TAG. Fatty acid oxidation from exogenous FA is regulated by DGAT activities. DAG, diacylglycerol; DGAT, diacylglycerol acyltransferase; FA, fatty acid; Glycerol-3-P, glycerol-3-phosphate; MAG, monoacylglycerol; TAG, triacylglycerol; TAG′ and TAG″, heterogeneous pools of TAG.

In this study we demonstrated how inhibitors of DGAT1 and DGAT2 had distinct responses on lipid metabolism in skeletal muscle cells. DGAT1 seemed to be the major enzyme responsible for incorporation of both exogenously supplied and endogenously generated (through lipolysis) FAs into cellular lipids in human myotubes, as well as being involved in *de novo* synthesis of TAG (Fig. 7). On the other hand, DGAT2 seemed mainly to be contributing in *de novo* synthesis of TAG from glycerol-3-phosphate. Interestingly, DGAT activities were also important in determining the rates of FA oxidation, indicating a key role in balancing FAs between storage in TAG and efficient utilization through oxidation (Fig. 7). Because DGAT1 and DGAT2 may have distinct roles, they could give rise to heterogeneous pools of TAG in skeletal muscle. However, more studies are required to determine and further elucidate the mechanism and possible beneficial/deleterious effects of DGAT1 and DGAT2 on energy metabolism in skeletal muscle.

## Supplementary information

Supplementary Information.

Supplementary Information2.

Supplementary Information3.

Supplementary Information4.
