# Supplementary figures and images for: Treatment of human skeletal muscle cells with inhibitors of diacylglycerol acyltransferases 1 and 2 to explore isozyme-specific roles on lipid metabolism
Source: Sci Rep. 2020 Jan 14;10:238. doi: 10.1038/s41598-019-57157-5 (PMC6959318; doi:10.1038/s41598-019-57157-5)

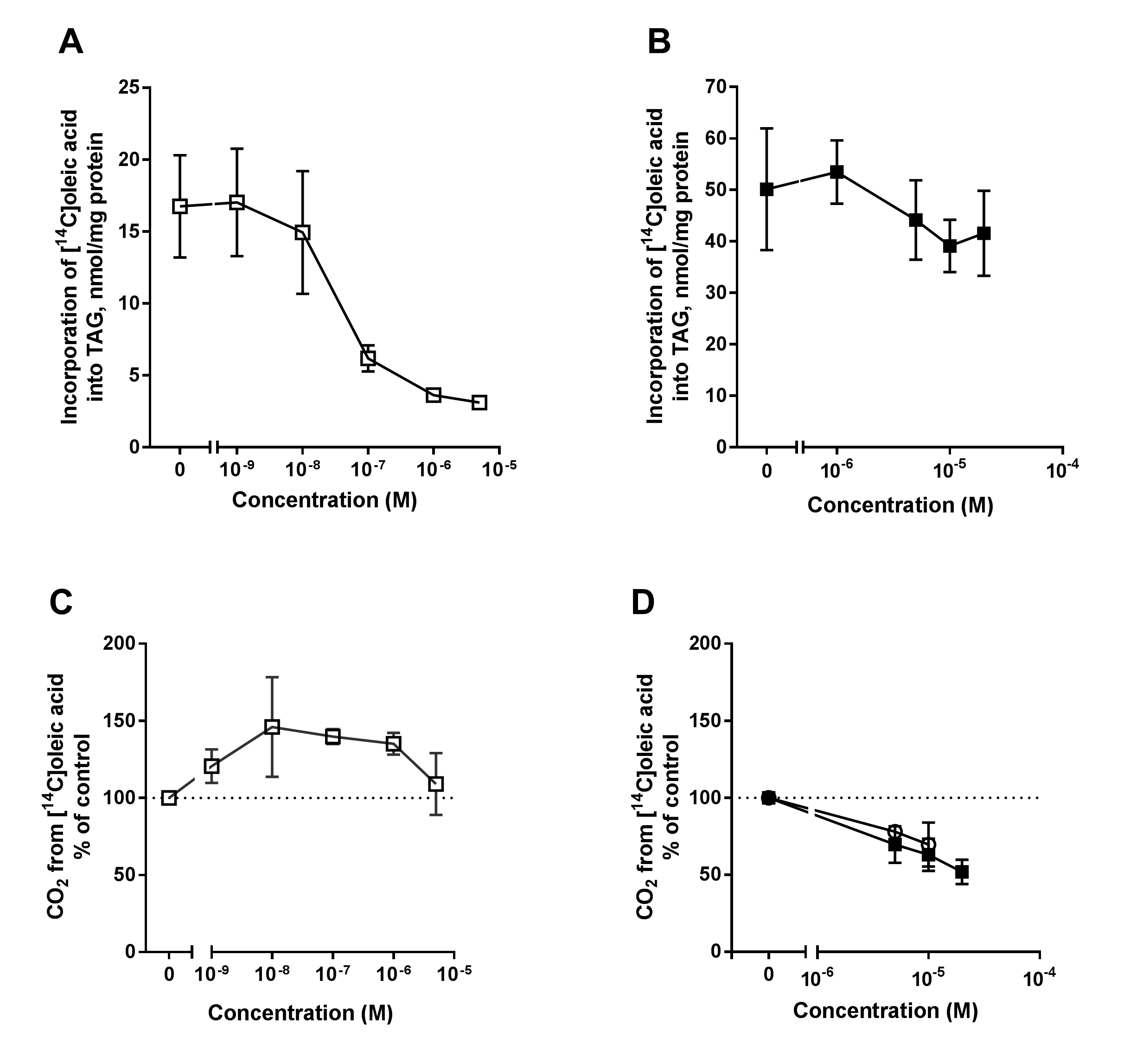

Supplement: Supplementary file 1 — Supplementary Information. [file 41598_2019_57157_MOESM1_ESM.tif]

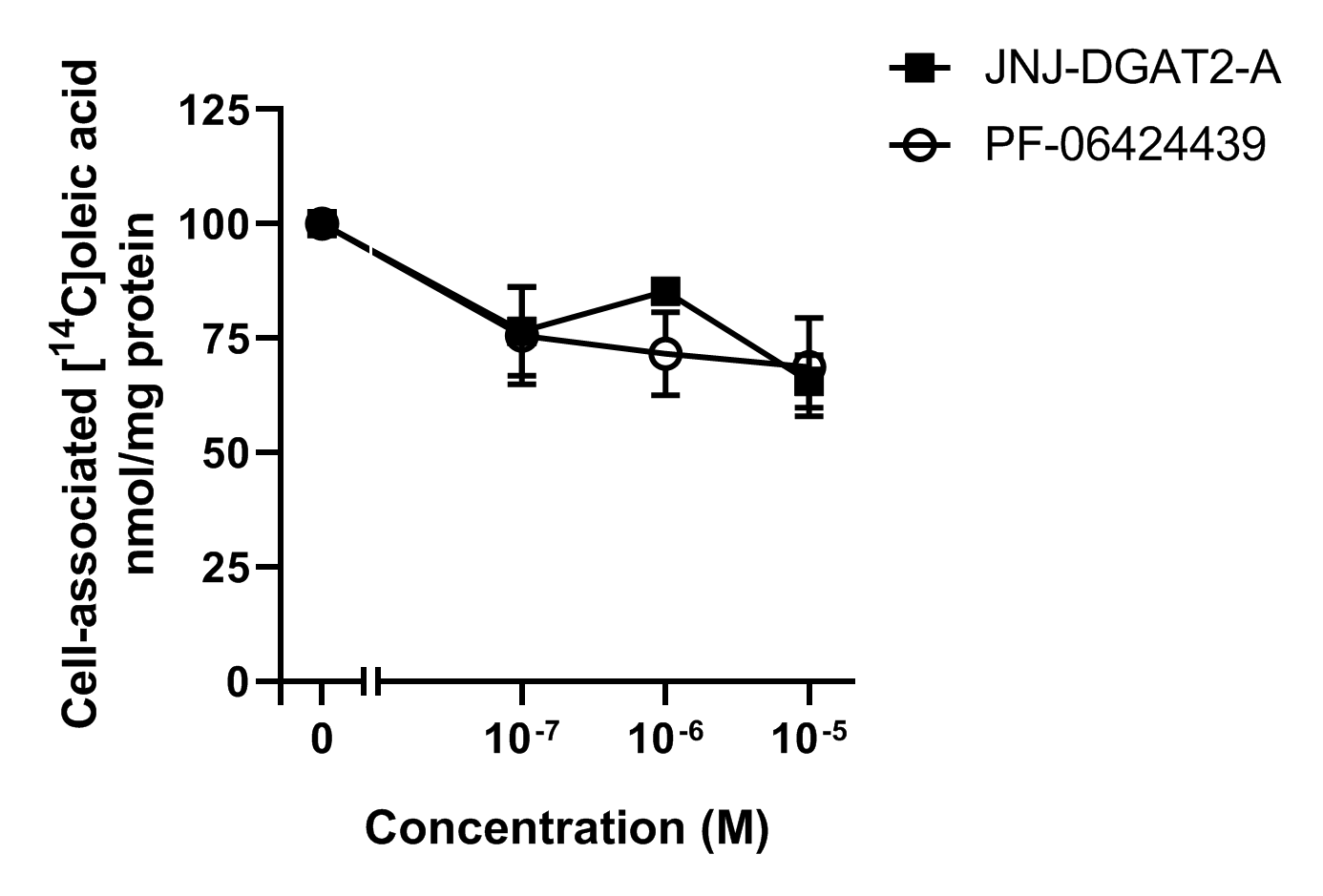

Supplement: Supplementary file 2 — Supplementary Information2. [file 41598_2019_57157_MOESM2_ESM.tif]

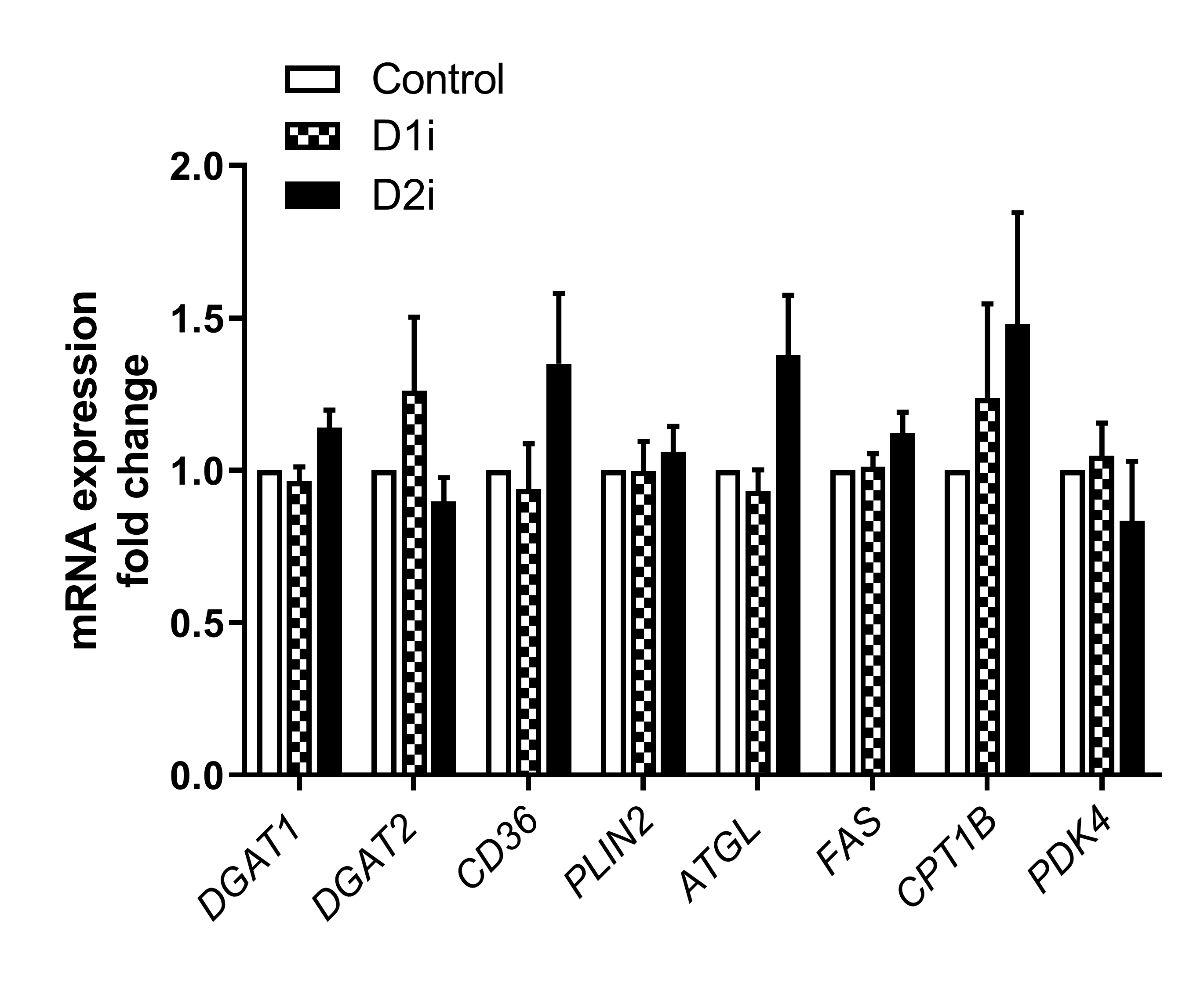

Supplement: Supplementary file 3 — Supplementary Information3. [file 41598_2019_57157_MOESM3_ESM.tif]

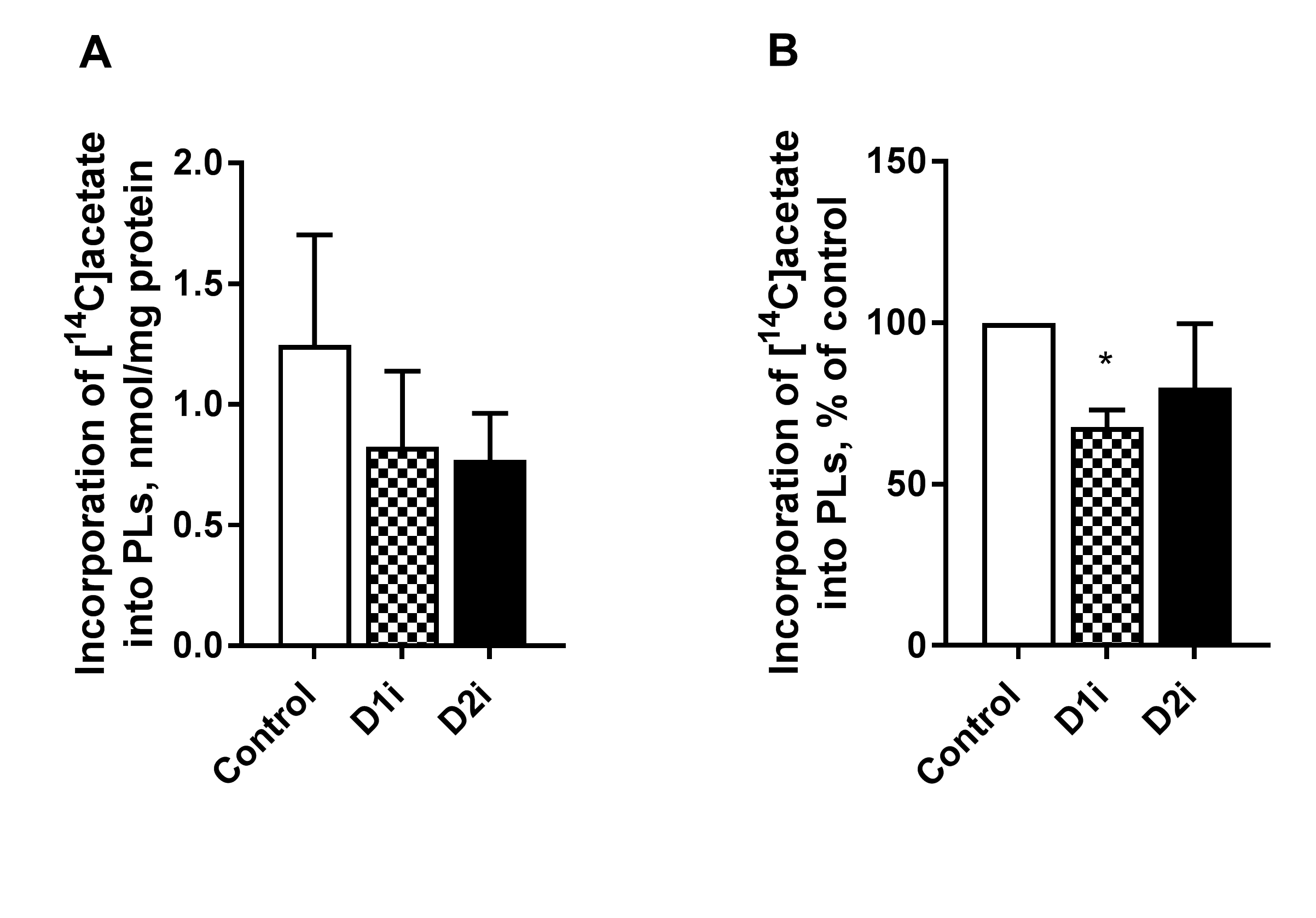

Supplement: Supplementary file 4 — Supplementary Information4. [file 41598_2019_57157_MOESM4_ESM.tif]
